# Dispensaries and Charitable Institutions of the Punjab

**Published:** 1901-12-07

**Authors:** 


### DISPENSARIES AND CHARITABLE INSTITUTIONS OF THE PUNJAB.

OF the 267 hospitals and dispensaries of all classes open on January 1st, 1899, in the Punjab, 263 remained at the end of the year, to which nine new ones had been added. Of these, seven were local fund institutions, and the other two private dispensaries, aided by local funds. The history of one of the closed dispensaries is worth reciting. Sukesar is the sanatorium of the Shahpur District, and during its occupation each year by the district officials it had been usual to open a dispensary for their use just for the time. As for some reason no request was made for its re-opening in 1899, the authorities wisely determined to save the expense. Of the other three institutions, one was found not to be doing useful work, and the other two were in unsuitable localities, and the attendance was not large enough to justify their continuance. The general condition of the Punjab medical institutions is decidedly satisfactory. Fifty-one thousand nine hundred and sixty-seven indoor patients were treated during 1899, compared with 49,068 in 1898, which shows an increase of 2,899. This is the more cheering because ever since 1894 the attendance has been steadily decreasing. The reasons which have been previously given for this decrease, viz. (1) that greater discrimination was exercised in the matter of admissions to hospital; (2) that dispensaries were temporarily closed on account of plague requirements; and (3) that civil surgeons were withdrawn from their charges for plague duty all existed in 1899, and in addition the year was a very healthy one. The increase, therefore, without undue straining, may legitimately be supposed to be due to the increasing popularity of those institutions which thus attracted so many more patients. Another fact which is calculated to give satisfaction to the authorities is that at Jullundur 75 per cent. of the patients paid for their own diet, and that the total cost of dieting last year in that hospital, notwithstanding the large increase in numbers, was only two-thirds of the amount expended in 1898. The very largest number of eye cases—448,382—which has ever been recorded in one year, were under treatment in 1899, and the successful treatment of so serious and distressing a condition as eye disease in its various forms cannot fail to specially impress persons living in such remote places as Afghanistan and Chitral, from which districts some of the patients came. Apart from the physical benefit these persons derive, much good accrues to them by being brought into contact with civilised humanity. One of the reasons cited for the high statistics of eye diseases during the 12 months ending December, 1899, is that it was a period of special heat, glare, and dust, but cataract cases were also numerous, a successful result being obtained by means of operation in 86 per cent. of the patients treated. The necessity for a new ophthalmic wing at the Mayo Hospital, Lahore, is very great, and it is a matter for regret that, owing to famine, the Government were unable to provide the funds. The overcrowding in this hospital is already considerable, so much so, that the verandahs on both sides of some of the wards have to be utilised for the accommodation of patients. The filling of the verandahs of a hospital ward with patients is opposed to all ideas of hospital sanitation, and must have an unfavourable influence on the health of those occupying the wards. A long grass shed with accommodation for 50 beds was also provided to receive the overflow from the hospital building. This shed was generally full of patients suffering from cataract. The authorities, however, issued orders for the discontinuance of the arrangement owing to the risk of fire, as a spark from a neighbouring building might set it in flames, and in that case few would escape. Occasionally, as many as 326 indoor patients have somehow been accommodated in the hospital, although the beds only number 149. There is little hope that the new wing will be built by private subscriptions, because, although the benefits which accrue from the hospital are fully recognised in Lahore, the amount given privately by the native community in 1899 only amounted to 144 rs., a most discreditably small sum. Elsewhere, however, native subscriptions have increased considerably, and much liberality has been shown. In Simla Mr. James Lewis Walker, an old and well-known resident, made a most generous offer of a property for the erection thereon of a European hospital, a much-felt want not only for Simla itself but also for European residents in the Punjab generally. The present Ripon Hospital is inconveniently situated for Europeans, and is also required for natives, who are being crowded out to find accommodation for the increasing number of Europeans resorting to the hospital for medical treatment. The Imperial and Provincial Governments have agreed to contribute towards the project, though it will be some time before the work approaches completion. There is still a great demand everywhere for female medical aid, but owing to the difficulty of finding suitable women willing to be trained many requests have to be refused and good work is left undone.

